# Crystal structure and UV spectra of a 1,2-disubstituted benzimidazolium chloride

**DOI:** 10.1107/S205698901700977X

**Published:** 2017-07-07

**Authors:** Tuhin Khan, Navneet Mishra, Darshan S. Mhatre, Anindya Datta

**Affiliations:** a Department of Chemistry, Indian Institute of Technology Bombay, Mumbai 400 076, India

**Keywords:** crystal structure, benzimidazole, hydrogen bonding, Hirshfeld surface analysis, DFT calculations, UV spectra

## Abstract

1-(2-Hy­droxy­benz­yl)-2-(2-hy­droxy­phen­yl)-1*H*-benzimidazol-3-ium chloride, C_20_H_17_N_2_O_2_^+^·Cl^−^, was prepared by reaction of salicyl­aldehyde with *o*-phenyl­enedi­amine in the presence of tri­methyl­silyl chloride acting as a source of HCl. As a result of steric hindrance, the cation in the crystal is far from planar: the benzimidazole ring system makes dihedral angles of 55.49 (9) and 81.36 (8)° with the planes of the phenolic groups. The crystal packing is dominated by O—H⋯Cl and N—H⋯Cl hydrogen bonds, which link the cations and anions into four-membered rings and then into chains along [100]. The title compound exhibits two transitions in the UV region, which are revealed in the solid state and solution spectra as an absorption maximum at 280 nm and a shoulder at 320 nm. According to the results of TD–DFT calculation, both transitions have a π–π* nature and the mol­ecular orbitals involved in these transitions are mostly localized on the benzimidazole ring system and on the phenyl ring attached to it at the 2-position.

## Abstract 2

1-(2-Hy­droxy­benz­yl)-2-(2-hy­droxy­phen­yl)-1*H*-benzimidazol-3-ium chloride, C_20_H_17_N_2_O_2_^+^·Cl^−^, is prepared by reaction of salicyl­aldehyde with *o*-phenyl­enedi­amine in the presence of tri­methyl­silyl chloride acting as a source of HCl. As a result of steric hindrance, the cation in the crystal is far from planar: the benzimidazole ring system makes dihedral angles of 55.49 (9) and 81.36 (8)° with the planes of the phenolic groups. The crystal packing is dominated by the O—H⋯Cl and N—H⋯Cl hydrogen bonds. The title compound exhibits two transitions in the UV region, which are revealed in the solid state and solution spectra as an absorption maximum at 280 nm and a shoulder at 320 nm. According to the results of TD–DFT calculation, both transitions have a π–π* nature and the mol­ecular orbitals involved in these transitions are mostly localized on the benzimidazole ring system and on the phenyl ring attached to it at the 2-position.

## Chemical context   

Benzimidazole derivatives are well known to exhibit anti­bacterial, anti­malarial and anti-inflammatory properties (Keri *et al.*, 2015 ▸; Carvalho *et al.*, 2011 ▸). Besides this, 1,2-disubstituted benzimidazoles are used as inter­mediates in synthesis of dyes and pigments (Carvalho *et al.*, 2011 ▸). Some substituted benzimidazoles, *e.g.* 2-(2′-hy­droxy­phen­yl)benzimidazole and its derivatives, are strongly fluorescent and show dual emission due to the excited state proton transfer (Douhal *et al.*, 1994 ▸). In the solid state, these compounds exhibit fluorescence, which is governed by their polymorphism and steric effects (Konoshima *et al.*, 2012 ▸; Benelhadj *et al.*, 2013 ▸; Shida *et al.*, 2013 ▸). Thus, this class of compounds is considered for applications in fluorescence imaging and optoelectronics (Zhao *et al.*, 2011 ▸). Benzimidazolium salts attract attention due to their non-linear optical properties (Sun *et al.*, 2011 ▸; Wang *et al.*, 2011 ▸). 2-(2′-Hy­droxy­phen­yl)benzimidazole, which is a member of this class of compounds, exhibits rotamerism (Ríos Vazquez *et al.*, 2008 ▸). In this work, the crystal structure of 1-(2-hy­droxy­benz­yl)-2-(2-hy­droxy­phen­yl)-1*H*-benzimidazol-3-ium chloride and its UV spectra have been reported. DFT calculations were carried out to study the geometry and electronic transitions.

## Structural commentary   

**Figure 1:**
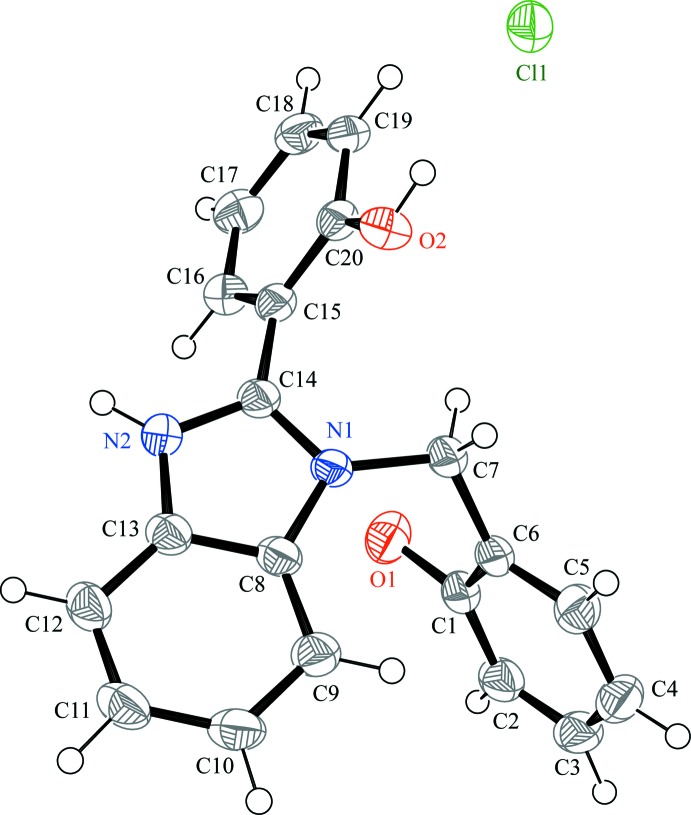
*ORTEP* diagram of the title compound with displacement ellipsoids drawn at the 50% probability level.

All bond lengths and bond angles are within the ranges reported for similar structures (Ha, 2012 ▸). The asymmetric unit, consisting of a 1-(2-hy­droxy­benz­yl)-2-(2-hy­droxy­phen­yl)-1*H*-benzo[*d*]imidazol-3-ium cation and a chloride anion, is presented in Fig. 1 ▸. As a result of steric hindrance, the cation is far from planar: the benzimidazole ring system makes dihedral angles of 55.49 (9) and 81.36 (8)° with the planes of phenolic groups immediately attached to it at position 2 and linked via the methyl­ene bridge to position 1, respectively. The deviation from planarity in the 2-(2-hy­droxy­phen­yl)benzimidazolium skeleton is larger than in the reported similar structures (Al-Douh *et al.*, 2009*b* ▸; Wang *et al.*, 2011 ▸).
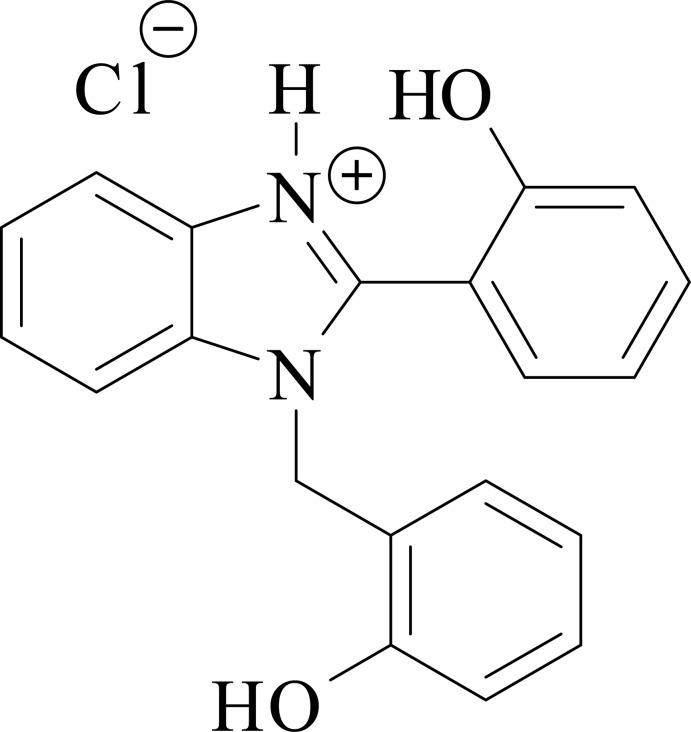


## Supra­molecular features   

**Figure 2:**
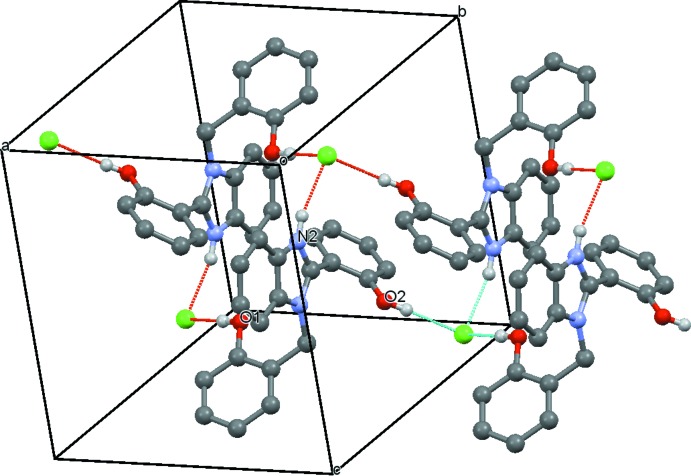
Packing diagram highlighting the hydrogen-bonding inter­actions.

In the crystal, each cation forms three hydrogen bonds, two O—H⋯Cl and one N—H⋯Cl (Table 1 ▸), to chloride anions. As a result of these inter­actions, the cations and anions form ribbons along [100], which consist of centrosymmetric four-membered rings each formed by two cations and two anions in the 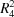
(16) and 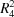
(20) manner, as shown in Fig. 2 ▸. Some weak contacts C—H⋯O, C—H⋯Cl and C—H⋯π are also present (Table 1 ▸).

## Hirshfeld surface analysis   

**Figure 3:**
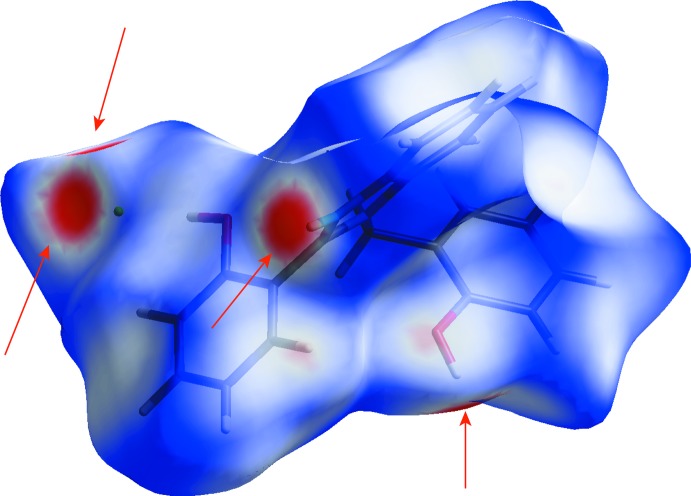
Hirshfeld surface of the ionic pair mapped with normalized contact distances (*d*_norm_) indicated by red spots. Positions of close contacts are highlighted by red arrows.

To evaluate the effect of close range inter­actions and compare their significance, Hirshfeld surface analysis (Spackman & Jayatilaka, 2009 ▸; Soman *et al.*, 2014 ▸) has been performed and its results are presented in Fig. 3 ▸. Four red spots on the Hirshfeld surface indicate short contacts. All close inter­actions are mediated by Cl^−^ anions. The H⋯H and C⋯H inter­actions are associated with 46% and 26% surface area, respectively. The contributions of the Cl⋯H (15%) and O⋯H (6%) inter­actions are smaller, but significant for the crystal architecture.

## Quantum chemical calculation   

The geometry of the cation–anion pair in the gas phase was optimized with density functional theory (DFT) using *GAUSSIAN09* package (Frisch *et al.*, 2009 ▸) within the framework of B3LYP/6-31G(d). Frequency calculations were carried out to confirm that the structure corresponds to a minimum. The optimized bond lengths agree with those observed in the crystal structure within the range of 0.04 Å (Table 2 ▸). The largest distinction between the calculated and crystallographic geometries is related to the twist of the phenolic group attached to the benzimidazole ring system at position 2: in the crystal, the corresponding torsion angles are by 7–14° nearer to 180° than the calculated values (Table 2 ▸). This could be due to the hydrogen-bonding and C—H⋯π inter­actions. The ionic nature of the optimized cation–anion pair is reflected in the large calculated dipole moment of 18.05 D. The time-dependent DFT (TD–DFT) calculation was performed on the crystal geometry at the same level of theory as for geometry optimization.

## UV spectra   

**Figure 4:**
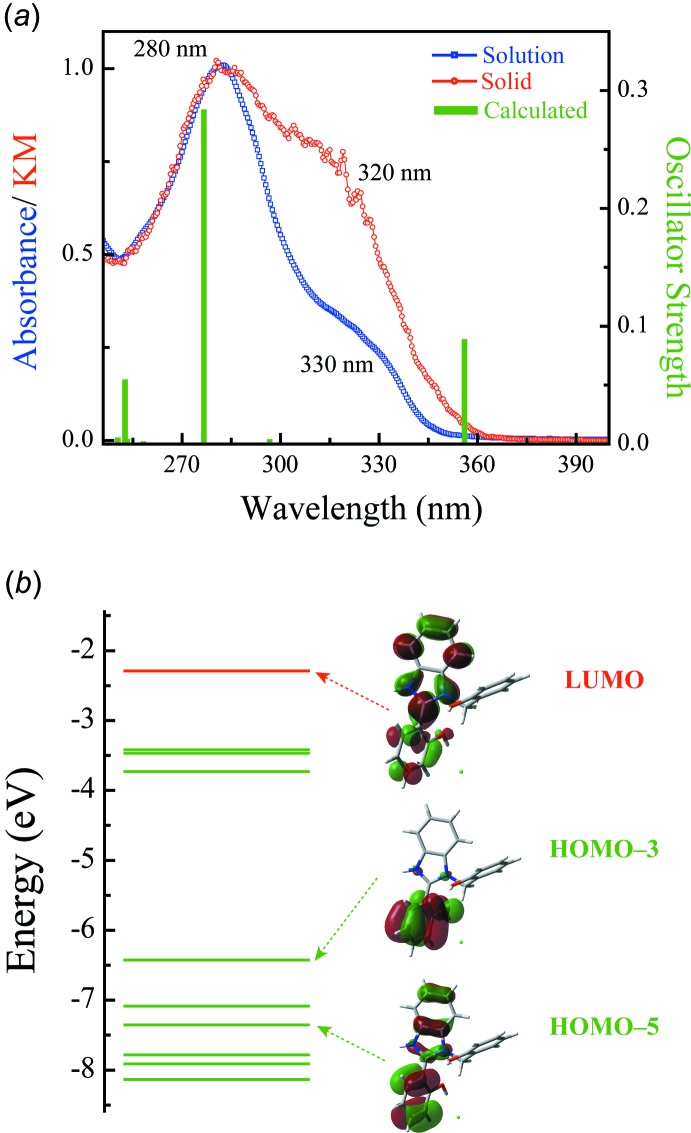
(*a*) Peak-normalized absorption spectrum of the compound in methanolic solution (blue), Kubelka–Munk (KM) transformed diffuse reflectance solid-state spectrum (red) and TD–DFT calculated transitions (green). (*b*) Mol­ecular orbital energy levels and the relevant Kohn–Sham orbitals.

The solid-state diffuse reflectance spectrum was measured with a Shimadzu-3600 spectrophotometer fitted with an MPC-3100 sample compartment. For that, the crystals were crushed to powder and mixed with BaSO_4_ to a final concentration of 5% (*v*/*v*). The Kubelka–Munk transformation (Kubelka & Munk, 1931 ▸) was applied to the reflectance data. The spectrum of methanol solution was measured with JASCO V530 spectrophotometer. The solid-state spectrum closely resembles the spectrum of the solution, thus indicating that the geometry and electronic structure of the cation did not change in moving from solid state to solution. In the UV region, the title compound exhibits an absorption maximum at 280 nm and a shoulder around 320 nm (Fig. 4 ▸ *a*). The absorption maximum at 280 nm is typical of benzimidazole (Hirayama, 1967 ▸), and the 320 nm shoulder is typical of benzimidazole derivatives (Mosquera *et al.*, 1996 ▸; Konoshima *et al.*, 2012 ▸). The Kubelka–Munk transformed spectrum of the solid sample is quite close to that of a structurally similar derivative reported earlier (Shida *et al.*, 2013 ▸). The positions and intensities of calculated transitions agree well with the experimental data (Fig. 4 ▸ *a*, Table 3 ▸). The transition at 277 nm is found to have the π–π* nature. The associated mol­ecular orbitals (HOMO-5 and LUMO) are spread over benzimidazole and 2-phenyl group (Fig. 4 ▸ *b*, Table 3 ▸). On the other hand, HOMO-3 is localized on 2-phenyl group, making the transitions at 356 nm partially charge-transfer in nature.

## Database survey   

A survey of Cambridge Structure Database (CSD version 5.36, November 2016) (Groom *et al.*, 2016 ▸) for mol­ecules with the 2-[1-(2-hy­droxy­benz­yl)-1*H*-benzo[*d*]imidazol-2-yl]phenol skeleton gave 18 hits. All of them are neutral mol­ecules. Among them are an *o*-methyl­ated derivative of the title compound (VIRZEC; Tarte *et al.*, 2007 ▸), an *o*-eth­oxy derivative (ZARFEF; Ha) , *o*-meth­oxy derivatives (VOQVAZ and VOQRUP; Al-Douh *et al.*, 2009*a* ▸ and Ha, 2012 ▸, respectively). Halide derivatives (CIQQOJ, NEGRIB) have also been reported (Fang *et al.*, 2007 ▸; Yang *et al.*, 2006 ▸). A search for protonated mol­ecules containing the 1-benzyl-2-phenyl-1*H*-benzo[*d*]imidazol-3-ium skeleton gave 11 hits, three of which being closely related to this work are reported in the same article (EBOHOU, EBOHUA and EBOJAI; Wang *et al.*, 2011 ▸).

## Synthesis and crystallization   

Salicyl­aldehyde (SD Fine Chemicals, Mumbai, India), *o*-phenyl­enedi­amine (Sigma– Aldrich, USA) and tri­methyl­silyl chloride (Sigma–Aldrich, USA) were used as received. The title compound was synthesized by the reaction of *o*-phenyl­enedi­amine (1 g) with salicyl­aldehyde (1:2 mole ratio) in double distilled water at 363 K using tri­methyl­silyl chloride as catalyst (1:1 molar ratio with respect to *o*-phenyl­enedi­amine) for 8–10 h (Wan *et al.*, 2009 ▸). The reaction mixture was cooled to room temperature, and the white precipitate was filtered off, washed with water, dried by pressing against filter paper and allowed to dry at ambient conditions over a few days. Unexpectedly, the product turned out to be a salt, not a neutral compound, as prescribed by the literature synthetic procedure. It was crystallized from a solution in aceto­nitrile/methanol mixture (15:85) in a refrigerator and then at room temperature. The resulting plate-shaped crystals were used for single crystal XRD measurements. Even after repeated attempts with crude and recrystallized samples, a clean ^1^H NMR spectrum, which is an indication of rotamerism in solution, was not obtained. For the spectroscopic study, the parent solvent was deca­nted and then the crystals were washed with diethyl ether and finally air dried.

## Refinement   

Crystal data, data collection and structure refinement details are summarized in Table 4 ▸. All H atoms were positioned geometrically (O—H = 0.84, N—H = 0.88, C—H = 0.95–0.99 Å)and refined using a riding model with *U*_iso_(H) = 1.2*U*_eq_(C,N) [1.5*U*_eq_(O)]. OH groups were allowed to rotate about the C—bonds.

## Supplementary Material

Crystal structure: contains datablock(s) I. DOI: 10.1107/S205698901700977X/yk2108sup1.cif

Structure factors: contains datablock(s) I. DOI: 10.1107/S205698901700977X/yk2108Isup2.hkl

Supporting information file. DOI: 10.1107/S205698901700977X/yk2108Isup3.cml

CCDC reference: 1548944

Additional supporting information:  crystallographic information; 3D view; checkCIF report

## Figures and Tables

**Table 1:** Hydrogen-bond geometry (Å, °)

*D*—H⋯*A*	*D*—H	H⋯*A*	*D*⋯*A*	*D*—H⋯*A*
O1—H1⋯Cl1^i^	0.84	2.24	3.066 (2)	169
O2—H2⋯Cl1	0.84	2.23	3.071 (1)	177
N2—H2*A*⋯Cl1^ii^	0.88	2.23	3.084 (2)	162
C16—H16⋯O1^iii^	0.95	2.57	3.253 (2)	129
C19—H19⋯Cl1	0.95	2.93	3.646 (2)	134
C7—H7*B*⋯*Cg*(C1-C6)^iv^	0.99	2.77	3.500 (2)	131

Symmetry codes: (i) 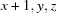; (ii) 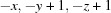; (iii) 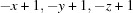; (iv) 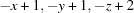.

**Table 2:** Comparison of notable bond lengths and torsion angles (Å, °)

	Crystal	DFT optimized
C20—O2	1.356 (2)	1.315
C20—C19	1.391 (3)	1.413
C14—N2	1.337 (2)	1.356
C14—N1	1.344 (2)	1.349
∠ C16—C15—C14—N1	123.4 (2)	137.99
∠ C20—C15—C14—N2	125.5 (2)	132.45

**Table 3:** Prominent electronic transitions obtained from TD–DFT calculation

Wavelength	Oscillator strength	Transition
356 nm	0.088	LUMO←HOMO-3 (98%)
277 nm	0.2827	LUMO←HOMO-5 (96%)
253 nm	0.0537	LUMO+2←HOMO-3 (78%)
		LUMO+3←HOMO-3 (12%)

**Table 4:** Experimental details

Crystal data	
Chemical formula	C_20_H_17_N_2_O_2_^+^·Cl^−^
*M*_r_	352.80
Crystal system, space group	Triclinic, *P* 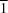
Temperature (K)	150
*a*, *b*, *c* (Å)	9.8002 (4), 10.6791 (5), 10.6986 (4)
α, β, γ (°)	111.364 (4), 102.346 (3), 111.311 (4)
*V* (Å^3^)	890.75 (7)
*Z*	2
Radiation type	Mo *K*α
μ (mm^−1^)	0.23
Crystal size (mm)	0.19 × 0.18 × 0.12

Data collection	
Diffractometer	Rigaku Saturn 724
Absorption correction	Multi-scan (*CrysAlis PRO*; Rigaku Oxford Diffraction, 2015 ▸)
*T*_min_, *T*_max_	0.657, 1.000
No. of measured, independent and observed [*I* > 2σ(*I*)] reflections	8813, 3114, 2654
*R*_int_	0.033
(sin θ/λ)_max_ (Å^−1^)	0.595

Refinement	
*R*[*F* ^2^ > 2σ(*F* ^2^)], *wR*(*F* ^2^), *S*	0.039, 0.105, 1.04
No. of reflections	3114
No. of parameters	228
H-atom treatment	H-atom parameters constrained
Δρ_max_, Δρ_min_ (e Å^−3^)	0.25, −0.21

Computer programs: *CrysAlis PRO* (Rigaku Oxford Diffraction, 2015 ▸), *SHELXT* (Sheldrick, 2015*a* ▸), *SHELXL2014* (Sheldrick, 2015*b* ▸) and *OLEX2* (Dolomanov *et al.*, 2009 ▸).

## supplementary crystallographic information

### Crystal data

**Table:**

C_20_H_17_N_2_O_2_^+^·Cl^−^	*Z* = 2
*M**_r_* = 352.80	*F*(000) = 368
Triclinic, *P*1	*D*_x_ = 1.315 Mg m^−^^3^
*a* = 9.8002 (4) Å	Mo *K*α radiation, λ = 0.71073 Å
*b* = 10.6791 (5) Å	Cell parameters from 5921 reflections
*c* = 10.6986 (4) Å	θ = 2.3–31.1°
α = 111.364 (4)°	µ = 0.23 mm^−^^1^
β = 102.346 (3)°	*T* = 150 K
γ = 111.311 (4)°	Plate, colourless
*V* = 890.75 (7) Å^3^	0.19 × 0.18 × 0.12 mm

### Data collection

**Table:**

Rigaku Saturn 724 diffractometer	3114 independent reflections
Radiation source: fine-focus sealed X-ray tube, Enhance (Mo) X-ray Source	2654 reflections with *I* > 2σ(*I*)
Graphite monochromator	*R*_int_ = 0.033
ω scans	θ_max_ = 25.0°, θ_min_ = 2.3°
Absorption correction: multi-scan (CrysAlis PRO; Rigaku Oxford Diffraction, 2015)	*h* = −11→10
*T*_min_ = 0.657, *T*_max_ = 1.000	*k* = −12→12
8813 measured reflections	*l* = −11→12

### Refinement

**Table:**

Refinement on *F*^2^	0 restraints
Least-squares matrix: full	Hydrogen site location: inferred from neighbouring sites
*R*[*F*^2^ > 2σ(*F*^2^)] = 0.039	H-atom parameters constrained
*wR*(*F*^2^) = 0.105	*w* = 1/[σ^2^(*F*_o_^2^) + (0.0483*P*)^2^ + 0.2976*P*] where *P* = (*F*_o_^2^ + 2*F*_c_^2^)/3
*S* = 1.04	(Δ/σ)_max_ = 0.001
3114 reflections	Δρ_max_ = 0.25 e Å^−^^3^
228 parameters	Δρ_min_ = −0.21 e Å^−^^3^

### Special details

**Table:**

Geometry. All esds (except the esd in the dihedral angle between two l.s. planes) are estimated using the full covariance matrix. The cell esds are taken into account individually in the estimation of esds in distances, angles and torsion angles; correlations between esds in cell parameters are only used when they are defined by crystal symmetry. An approximate (isotropic) treatment of cell esds is used for estimating esds involving l.s. planes.
Refinement. 1. Fixed Uiso At 1.2 times of: All C(H) groups, All C(H,H) groups, All N(H) groups At 1.5 times of: All O(H) groups 2.a Secondary CH2 refined with riding coordinates: C7(H7A,H7B) 2.b Aromatic/amide H refined with riding coordinates: N2(H2A), C2(H2B), C3(H3), C4(H4), C5(H5), C9(H9), C10(H10), C11(H11), C12(H12), C16(H16), C17(H17), C18(H18), C19(H19) 2.c Idealised tetrahedral OH refined as rotating group: O1(H1), O2(H2)

### Fractional atomic coordinates and isotropic or equivalent isotropic displacement parameters (Å^2^)

**Table:**

	*x*	*y*	*z*	*U*_iso_*/*U*_eq_	
Cl1	−0.06343 (6)	0.67366 (6)	0.86942 (5)	0.04152 (17)	
O1	0.57838 (18)	0.45573 (18)	0.75661 (16)	0.0457 (4)	
H1	0.6774	0.5046	0.7816	0.069*	
O2	0.07420 (16)	0.49890 (14)	0.68652 (15)	0.0381 (3)	
H2	0.0365	0.5481	0.7349	0.057*	
N1	0.23361 (18)	0.29524 (16)	0.60618 (15)	0.0302 (3)	
N2	0.18508 (19)	0.27540 (17)	0.38883 (16)	0.0337 (4)	
H2A	0.1727	0.2994	0.3183	0.040*	
C7	0.2833 (2)	0.3651 (2)	0.76670 (19)	0.0325 (4)	
H7A	0.1877	0.3293	0.7888	0.039*	
H7B	0.3331	0.4783	0.8101	0.039*	
C1	0.5475 (2)	0.3725 (2)	0.8279 (2)	0.0355 (4)	
C2	0.6572 (3)	0.3358 (2)	0.8901 (2)	0.0428 (5)	
H2B	0.7577	0.3671	0.8826	0.051*	
C3	0.6179 (3)	0.2535 (2)	0.9626 (2)	0.0467 (5)	
H3	0.6913	0.2262	1.0033	0.056*	
C4	0.4745 (3)	0.2105 (2)	0.9770 (2)	0.0467 (5)	
H4	0.4506	0.1571	1.0303	0.056*	
C5	0.3645 (3)	0.2453 (2)	0.9129 (2)	0.0400 (5)	
H5	0.2647	0.2148	0.9217	0.048*	
C6	0.3999 (2)	0.3248 (2)	0.83576 (18)	0.0325 (4)	
C8	0.1953 (2)	0.1454 (2)	0.50925 (19)	0.0321 (4)	
C9	0.1862 (2)	0.0217 (2)	0.5291 (2)	0.0380 (5)	
H9	0.2058	0.0277	0.6225	0.046*	
C10	0.1472 (3)	−0.1105 (2)	0.4064 (2)	0.0428 (5)	
H10	0.1417	−0.1968	0.4163	0.051*	
C11	0.1156 (3)	−0.1214 (2)	0.2682 (2)	0.0422 (5)	
H11	0.0886	−0.2150	0.1867	0.051*	
C12	0.1225 (2)	−0.0002 (2)	0.2470 (2)	0.0384 (5)	
H12	0.0995	−0.0078	0.1528	0.046*	
C13	0.1650 (2)	0.1346 (2)	0.3711 (2)	0.0333 (4)	
C14	0.2263 (2)	0.3700 (2)	0.52966 (19)	0.0309 (4)	
C15	0.2661 (2)	0.5309 (2)	0.58492 (19)	0.0319 (4)	
C16	0.3810 (2)	0.6224 (2)	0.5524 (2)	0.0385 (5)	
H16	0.4303	0.5785	0.4957	0.046*	
C17	0.4234 (3)	0.7744 (2)	0.6010 (2)	0.0428 (5)	
H17	0.5025	0.8362	0.5796	0.051*	
C18	0.3493 (3)	0.8366 (2)	0.6819 (2)	0.0402 (5)	
H18	0.3796	0.9423	0.7175	0.048*	
C19	0.2325 (2)	0.7481 (2)	0.7116 (2)	0.0356 (4)	
H19	0.1812	0.7922	0.7651	0.043*	
C20	0.1895 (2)	0.5940 (2)	0.66327 (19)	0.0314 (4)	

### Atomic displacement parameters (Å^2^)

**Table:**

	*U*^11^	*U*^22^	*U*^33^	*U*^12^	*U*^13^	*U*^23^
Cl1	0.0437 (3)	0.0520 (3)	0.0327 (3)	0.0275 (3)	0.0155 (2)	0.0196 (2)
O1	0.0415 (9)	0.0639 (10)	0.0441 (8)	0.0290 (8)	0.0202 (7)	0.0329 (8)
O2	0.0398 (8)	0.0354 (7)	0.0450 (8)	0.0232 (7)	0.0205 (7)	0.0183 (6)
N1	0.0339 (9)	0.0275 (8)	0.0257 (8)	0.0183 (7)	0.0072 (7)	0.0089 (6)
N2	0.0394 (10)	0.0352 (8)	0.0272 (8)	0.0214 (8)	0.0109 (7)	0.0139 (7)
C7	0.0370 (11)	0.0326 (10)	0.0250 (9)	0.0199 (9)	0.0095 (8)	0.0099 (8)
C1	0.0420 (12)	0.0379 (10)	0.0254 (9)	0.0232 (9)	0.0114 (9)	0.0117 (8)
C2	0.0449 (13)	0.0488 (12)	0.0330 (11)	0.0305 (11)	0.0106 (10)	0.0131 (9)
C3	0.0574 (15)	0.0436 (12)	0.0340 (11)	0.0329 (11)	0.0055 (10)	0.0127 (10)
C4	0.0639 (16)	0.0346 (11)	0.0321 (11)	0.0226 (11)	0.0068 (11)	0.0154 (9)
C5	0.0452 (13)	0.0335 (10)	0.0313 (10)	0.0161 (9)	0.0094 (9)	0.0126 (9)
C6	0.0401 (11)	0.0295 (9)	0.0217 (9)	0.0192 (9)	0.0073 (8)	0.0070 (8)
C8	0.0312 (11)	0.0289 (9)	0.0292 (10)	0.0172 (8)	0.0066 (8)	0.0079 (8)
C9	0.0400 (12)	0.0343 (10)	0.0343 (10)	0.0206 (9)	0.0074 (9)	0.0131 (9)
C10	0.0435 (13)	0.0312 (10)	0.0451 (12)	0.0222 (10)	0.0078 (10)	0.0117 (9)
C11	0.0391 (12)	0.0329 (10)	0.0366 (11)	0.0190 (9)	0.0069 (9)	0.0027 (9)
C12	0.0364 (12)	0.0385 (11)	0.0278 (10)	0.0192 (9)	0.0064 (9)	0.0067 (8)
C13	0.0291 (10)	0.0337 (10)	0.0317 (10)	0.0178 (9)	0.0079 (8)	0.0106 (8)
C14	0.0293 (10)	0.0332 (10)	0.0291 (9)	0.0187 (8)	0.0084 (8)	0.0123 (8)
C15	0.0339 (11)	0.0327 (10)	0.0278 (9)	0.0195 (9)	0.0072 (8)	0.0130 (8)
C16	0.0437 (12)	0.0436 (11)	0.0380 (11)	0.0276 (10)	0.0184 (10)	0.0216 (9)
C17	0.0440 (13)	0.0415 (11)	0.0499 (12)	0.0220 (10)	0.0189 (11)	0.0274 (10)
C18	0.0467 (13)	0.0311 (10)	0.0404 (11)	0.0203 (10)	0.0094 (10)	0.0181 (9)
C19	0.0418 (12)	0.0365 (10)	0.0326 (10)	0.0262 (10)	0.0113 (9)	0.0157 (9)
C20	0.0313 (10)	0.0340 (10)	0.0278 (9)	0.0178 (9)	0.0073 (8)	0.0145 (8)

### Geometric parameters (Å, º)

**Table:**

O1—H1	0.8400	C5—C6	1.393 (3)
O1—C1	1.365 (2)	C8—C9	1.388 (3)
O2—H2	0.8400	C8—C13	1.396 (3)
O2—C20	1.356 (2)	C9—H9	0.9500
N1—C7	1.479 (2)	C9—C10	1.380 (3)
N1—C8	1.402 (2)	C10—H10	0.9500
N1—C14	1.344 (2)	C10—C11	1.396 (3)
N2—H2A	0.8800	C11—H11	0.9500
N2—C13	1.378 (2)	C11—C12	1.374 (3)
N2—C14	1.337 (2)	C12—H12	0.9500
C7—H7A	0.9900	C12—C13	1.394 (2)
C7—H7B	0.9900	C14—C15	1.458 (2)
C7—C6	1.505 (2)	C15—C16	1.400 (3)
C1—C2	1.393 (3)	C15—C20	1.396 (3)
C1—C6	1.381 (3)	C16—H16	0.9500
C2—H2B	0.9500	C16—C17	1.371 (3)
C2—C3	1.380 (3)	C17—H17	0.9500
C3—H3	0.9500	C17—C18	1.385 (3)
C3—C4	1.373 (3)	C18—H18	0.9500
C4—H4	0.9500	C18—C19	1.375 (3)
C4—C5	1.391 (3)	C19—H19	0.9500
C5—H5	0.9500	C19—C20	1.391 (3)
			
C1—O1—H1	109.5	C10—C9—C8	116.71 (18)
C20—O2—H2	109.5	C10—C9—H9	121.6
C8—N1—C7	126.87 (14)	C9—C10—H10	118.9
C14—N1—C7	124.73 (14)	C9—C10—C11	122.14 (18)
C14—N1—C8	108.30 (14)	C11—C10—H10	118.9
C13—N2—H2A	125.2	C10—C11—H11	119.2
C14—N2—H2A	125.2	C12—C11—C10	121.63 (17)
C14—N2—C13	109.54 (15)	C12—C11—H11	119.2
N1—C7—H7A	109.2	C11—C12—H12	121.8
N1—C7—H7B	109.2	C11—C12—C13	116.42 (18)
N1—C7—C6	112.25 (14)	C13—C12—H12	121.8
H7A—C7—H7B	107.9	N2—C13—C8	106.63 (15)
C6—C7—H7A	109.2	N2—C13—C12	131.29 (17)
C6—C7—H7B	109.2	C12—C13—C8	122.08 (17)
O1—C1—C2	122.67 (19)	N1—C14—C15	128.09 (16)
O1—C1—C6	116.49 (16)	N2—C14—N1	109.17 (15)
C6—C1—C2	120.84 (18)	N2—C14—C15	122.61 (15)
C1—C2—H2B	120.4	C16—C15—C14	118.21 (16)
C3—C2—C1	119.1 (2)	C20—C15—C14	122.35 (17)
C3—C2—H2B	120.4	C20—C15—C16	119.39 (17)
C2—C3—H3	119.5	C15—C16—H16	119.5
C4—C3—C2	121.02 (19)	C17—C16—C15	120.94 (18)
C4—C3—H3	119.5	C17—C16—H16	119.5
C3—C4—H4	120.2	C16—C17—H17	120.5
C3—C4—C5	119.60 (19)	C16—C17—C18	119.01 (19)
C5—C4—H4	120.2	C18—C17—H17	120.5
C4—C5—H5	119.8	C17—C18—H18	119.4
C4—C5—C6	120.3 (2)	C19—C18—C17	121.23 (18)
C6—C5—H5	119.8	C19—C18—H18	119.4
C1—C6—C7	119.68 (16)	C18—C19—H19	120.0
C1—C6—C5	119.00 (18)	C18—C19—C20	120.08 (17)
C5—C6—C7	121.27 (18)	C20—C19—H19	120.0
C9—C8—N1	132.62 (17)	O2—C20—C15	117.13 (16)
C9—C8—C13	121.01 (16)	O2—C20—C19	123.57 (16)
C13—C8—N1	106.36 (15)	C19—C20—C15	119.29 (17)
C8—C9—H9	121.6		
			
O1—C1—C2—C3	178.87 (18)	C8—C9—C10—C11	−1.1 (3)
O1—C1—C6—C7	0.3 (2)	C9—C8—C13—N2	−179.52 (17)
O1—C1—C6—C5	−177.18 (16)	C9—C8—C13—C12	0.8 (3)
N1—C7—C6—C1	63.1 (2)	C9—C10—C11—C12	0.4 (3)
N1—C7—C6—C5	−119.49 (18)	C10—C11—C12—C13	0.9 (3)
N1—C8—C9—C10	−178.6 (2)	C11—C12—C13—N2	178.91 (19)
N1—C8—C13—N2	−0.2 (2)	C11—C12—C13—C8	−1.5 (3)
N1—C8—C13—C12	−179.86 (17)	C13—N2—C14—N1	−0.2 (2)
N1—C14—C15—C16	123.4 (2)	C13—N2—C14—C15	175.93 (16)
N1—C14—C15—C20	−59.2 (3)	C13—C8—C9—C10	0.5 (3)
N2—C14—C15—C16	−51.9 (3)	C14—N1—C7—C6	−134.04 (18)
N2—C14—C15—C20	125.5 (2)	C14—N1—C8—C9	179.3 (2)
C7—N1—C8—C9	2.7 (3)	C14—N1—C8—C13	0.1 (2)
C7—N1—C8—C13	−176.58 (17)	C14—N2—C13—C8	0.2 (2)
C7—N1—C14—N2	176.79 (16)	C14—N2—C13—C12	179.9 (2)
C7—N1—C14—C15	1.0 (3)	C14—C15—C16—C17	179.90 (18)
C1—C2—C3—C4	−1.4 (3)	C14—C15—C20—O2	−0.1 (3)
C2—C1—C6—C7	−179.56 (16)	C14—C15—C20—C19	−179.40 (17)
C2—C1—C6—C5	3.0 (3)	C15—C16—C17—C18	−0.8 (3)
C2—C3—C4—C5	2.4 (3)	C16—C15—C20—O2	177.20 (16)
C3—C4—C5—C6	−0.6 (3)	C16—C15—C20—C19	−2.1 (3)
C4—C5—C6—C7	−179.43 (17)	C16—C17—C18—C19	−1.2 (3)
C4—C5—C6—C1	−2.0 (3)	C17—C18—C19—C20	1.6 (3)
C6—C1—C2—C3	−1.3 (3)	C18—C19—C20—O2	−179.12 (17)
C8—N1—C7—C6	42.1 (2)	C18—C19—C20—C15	0.1 (3)
C8—N1—C14—N2	0.1 (2)	C20—C15—C16—C17	2.5 (3)
C8—N1—C14—C15	−175.76 (18)		

### Hydrogen-bond geometry (Å, º)

**Table:**

*D*—H···*A*	*D*—H	H···*A*	*D*···*A*	*D*—H···*A*
O1—H1···Cl1^i^	0.84	2.24	3.066 (2)	169
O2—H2···Cl1	0.84	2.23	3.071 (1)	177
N2—H2*A*···Cl1^ii^	0.88	2.23	3.084 (2)	162
C16—H16···O1^iii^	0.95	2.57	3.253 (2)	129
C19—H19···Cl1	0.95	2.93	3.646 (2)	134
C7—H7*B*···*Cg*(C1-C6)^iv^	0.99	2.77	3.500 (2)	131

Symmetry codes: (i) *x*+1, *y*, *z*; (ii) −*x*, −*y*+1, −*z*+1; (iii) −*x*+1, −*y*+1, −*z*+1; (iv) −*x*+1, −*y*+1, −*z*+2.
